# An Unusual Case of Moraxella osleonsis Bacteremia in an Immunocompetent Patient With SARS-CoV-2 Infection

**DOI:** 10.7759/cureus.10154

**Published:** 2020-08-31

**Authors:** Bala C Veerabathini, Kaushik Manthani, Sandeep Gandhi

**Affiliations:** 1 Family Medicine, Peconic Bay Medical Center-Northwell Health, Riverhead, USA; 2 Infectious Disease, Peconic Bay Medical Center-Northwell Health, Riverhead, USA

**Keywords:** bacteremia, covid-19, coronavirus, moraxella, immunocompetent, immunocompromised, cd-4, cd-8, t cells, regulatory t cells

## Abstract

*Moraxella osleonsis *(*M.osleonsis *) is an organism that rarely presents with bacteremia in immunocompetent patients. We report a case of an immunocompetent 59-year-old male with a recent SARS-CoV-2 infection that developed *M.osleonsis* bacteremia. We believe that SARS-CoV-2 infection may have played a role in developing *M.osleonsis *bacteremia in this patient and may be one of the first reported cases of such bacteremia in a COVID-19 patient.

## Introduction

The species *Moraxella osleonsis *(*M.osleonsis*), first described in the literature in 1967, consists of aerobic gram-negative oxidase-positive coccobacilli. In humans, species of the *M.osleonsis* were found to be inhabitants of the upper respiratory tract [1]. This bacterium has been found in healthy adults’ nasopharynx, nose, and oropharynx [1-3]. However, individual reports of infection by this bacterium are rare and, as a result, M.osleonsis has been rarely studied for its clinical significance [1]. It has been reported to cause the following infections: septic arthritis, vaginitis, endocarditis, bacteremia, meningitis, and sinusitis [4-6]. 

The current coronavirus disease (COVID-19) pandemic caused by the severe acute respiratory syndrome coronavirus 2 (SARS-CoV-2) has emerged to be a cause for many ailments in humans. It is primarily known to affect the respiratory system but also causes cardiovascular disease, renal failure, and sequelae of a hypercoagulable state [7-9]. Being the first novel virus to have affected the human race in recent history, various mechanisms have been recently proposed to explain its effects. While the specific mechanisms are not fully understood, it is becoming more evident that a SARS-CoV-2 infection may be making humans more susceptible to various systemic diseases, including the possibility of causing rare bacteremia. 

## Case presentation

A 59-year-old male with a past medical history of Crohn’s disease, hypertension, hyperlipidemia, chronic obstructive pulmonary disease, bipolar disorder, and chronic back pain presented to the hospital with lethargy and vomiting. He had cough, nausea, vomiting and chronic back pain, but he denied chills, headaches, sore throat, diarrhea, loss of taste or smell, shortness of breath and muscle aches. Of note, he had a recent hospitalization for COVID-19 pneumonia two months prior where he was treated with ten days of hydroxychloroquine and five days of azithromycin. The patient reported no residual symptoms from his prior SARS-CoV-2 infection.

Upon admission, the patient reported that he had taken seven unspecified muscle relaxants to help alleviate his back pain. His home medications are listed in Table 1. Home medications were stopped on admission. He was not on any medications for Crohn's disease as he had no symptoms of the disease.

**Table 1:** Home Medications

Medication:	Dosage:	Frequency:
Valproic acid	250 mg	Three times daily
Clonazepam	1 mg	Once daily
Olanzapine	5 mg	Twice daily
Trazadone	100 mg	Once daily
Gabapentin	400 mg	Twice daily
Amlodipine	2.5 mg	Once daily
Medical marijuana	Unspecified	Unspecified

He appeared well-nourished, cooperative, and in no acute distress. He was conscious, alert and oriented to person, place and time, but seemed lethargic. The physical exam was significant for poor dentition but otherwise unremarkable, including a benign abdominal exam and a nonfocal neurological examination. Initial vitals are listed in Table 2 and pertinent lab results on admission are listed in Table 3. COVID-19 PCR test was positive for antigen and COVID-19 IgG antibody test was positive as well. Urine toxicology was positive for benzodiazepines and cannabinoids. 

**Table 2:** Initial Vital Signs

Vital sign:	Value:
Temperature	98.5° F
Blood pressure	108/71 mm Hg
Pulse	69 beats/min
Respiratory rate	16/min
Oxygen saturation	100% on room air

**Table 3:** Lab Results

	Lab value:	Normal range:
White blood cell (WBC) count	4,000/mm^3^	4,500 to 11,000/mm^3^
Neutrophils	80.4%	54 to 62%
Valproic acid level	140.90 ug/mL	50 to 100 ug/mL
Ammonia	49.7 µ/dL	15 to 45 µ/dL
Blood urea nitrogen (BUN)	19 mg/dL	7 to 20 mg/dL
Creatinine	1 mg/dL	.6 to 1.2 mg/dL
Total protein	6.3 g/dL	6.0 to 7.8 g/dL
Albumin	3.7 g/dL	3.5 to 5.5 g/dL
Alkaline phosphatase (ALP)	43 U/L	30 to 100 U/L
Aspartate aminotransferase (AST)	12 U/L	8 to 40 U/L
Alanine aminotransferase (ALT)	<5 U/L	8 to 40 U/L
COVID-19 IgG antibody level	18.9 g/L	<1 g/L

His initial infectious disease workup on day one was negative. On day two, one blood culture became positive for *M.osleonsis *and *Streptococcus* species (*salivarius*, *vestibularis*). He was started on 1 gram of intravenous ceftriaxone daily for 14 days, and repeat blood cultures on day four revealed no growth.

**Figure 1:**
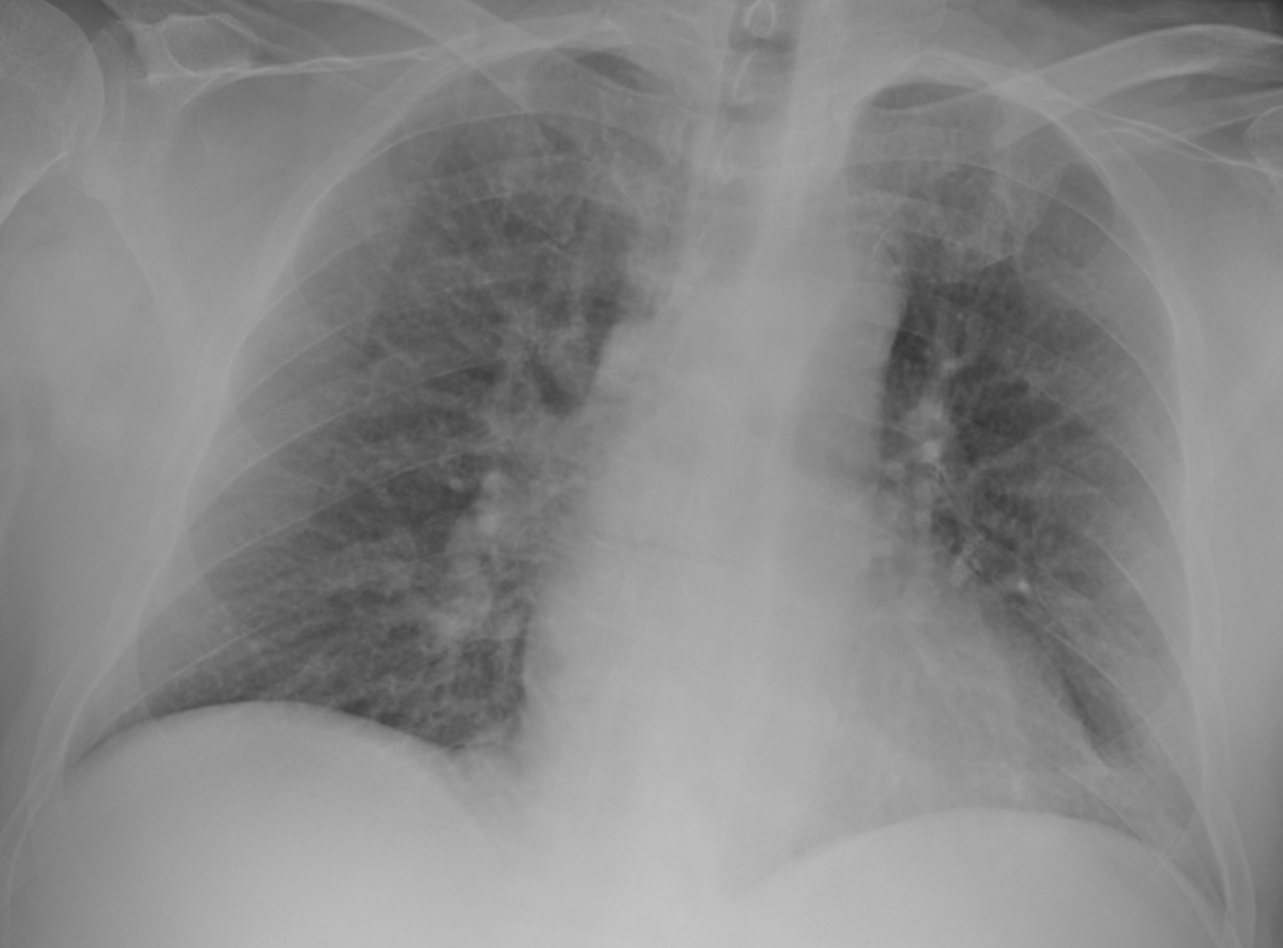
X-ray of the Chest (coronal view)

**Figure 2:**
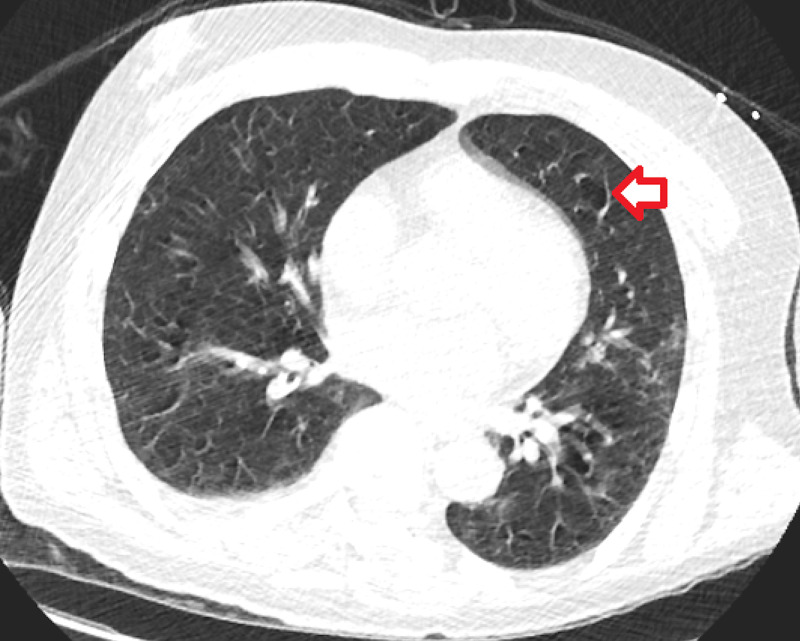
Computed Tomography of the Chest Without Contrast (axial view) Leftwards facing red arrow shows the emphysematous changes.

**Figure 3:**
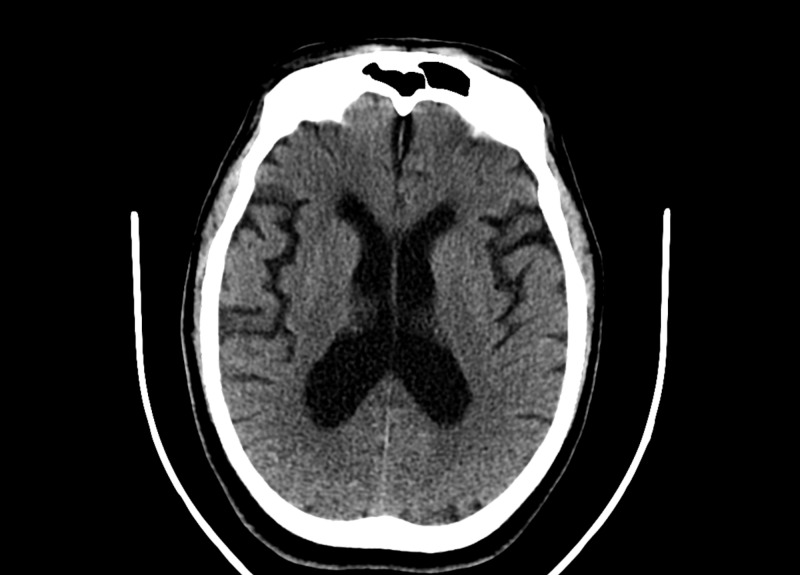
Computed Tomography of the Brain Without Contrast (axial view)

**Figure 4:**
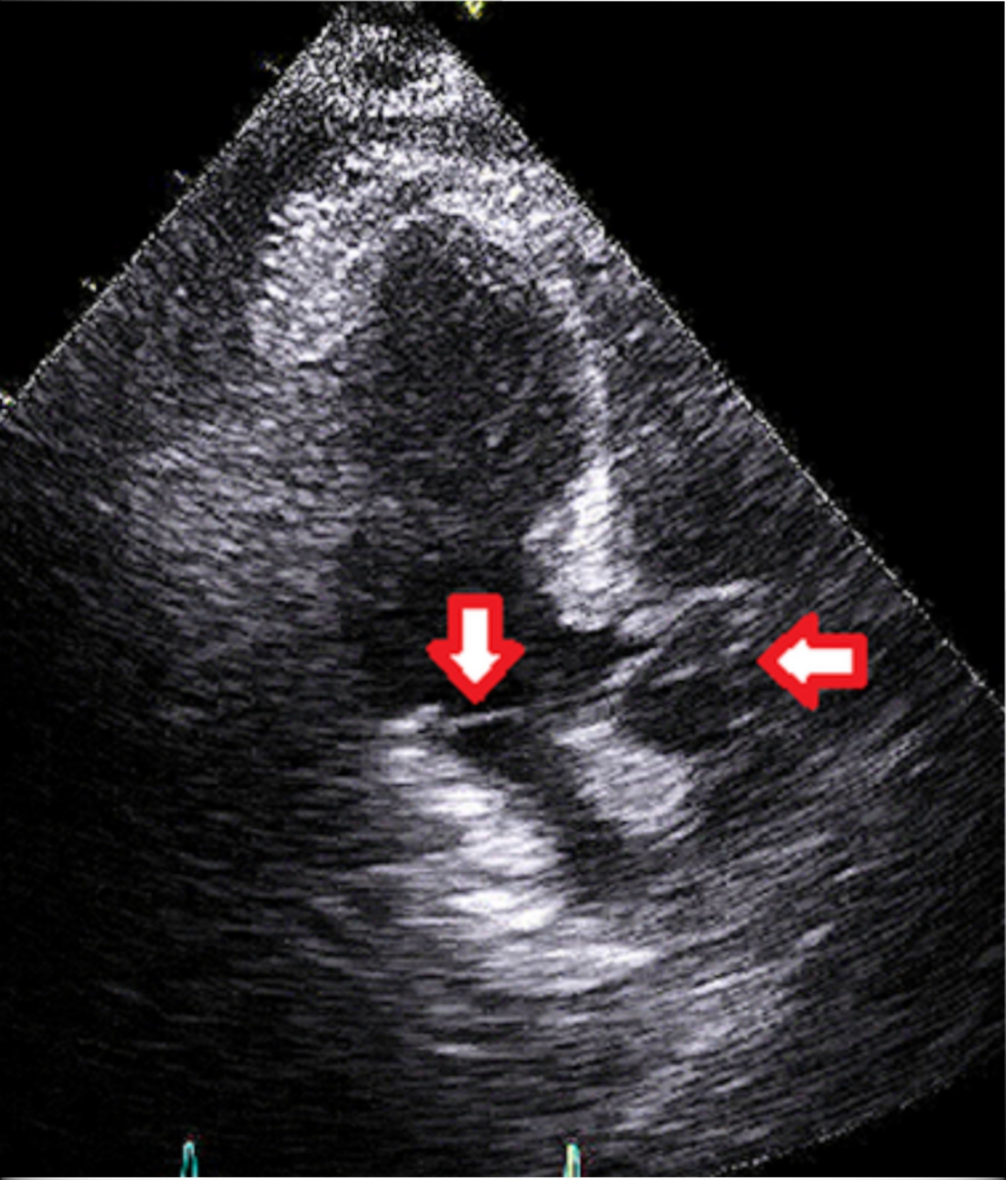
Transthoracic Echocardiogram (apical three-chamber view) Downwards facing red arrow shows the mitral valve, without any vegetations. Leftwards facing red arrow shows the aortic valve, also without vegetations.

The following imaging studies were done. A chest X-ray showed no acute cardiopulmonary disease findings (Figure 1). CT of the chest showed mild right apical paraseptal and centrilobular emphysematous change and dependent atelectatic changes at lung bases (Figure 2). CT of the brain showed no acute intracranial hemorrhage or mass effect from vasogenic edema (Figure 3). Transthoracic echocardiogram showed no vegetations with a left ventricular ejection fraction of 60% (Figure 4).

As mentioned, he continued intravenous ceftriaxone throughout his hospital stay. Valproic acid was restarted on day three once the valproic acid level was therapeutic. His encephalopathy improved drastically over time and the patient was discharged on day six. On discharge, he was prescribed an eighth-day course of intravenous ceftriaxone for completion of the antibiotic regimen. Dental evaluation, infectious disease, and psychiatry appointments were scheduled for outpatient follow-ups. 

## Discussion

Polypharmacy and medication side effects are an important consideration when developing differentials for patient symptoms. This patient was taking several potent psychiatric medications, including valproic acid, clonazepam, trazodone, and olanzapine, which can cause lethargy, encephalopathy, and vomiting [10-12]. Despite normal kidney and liver function tests, hyperammonemia was present in this patient due to supratherapeutic valproic acid levels. Symptoms of valproic acid toxicity can result in central nervous system depression, respiratory depression, metabolic abnormalities, nausea, vomiting, diarrhea, miosis, agitation, tremors, and myoclonus. Symptoms of hyperammonemia can include vomiting, ataxia, behavioral changes, lethargy, somnolence, and coma [12].

Beyond medication-related encephalopathy, other etiologies were sought, including bacteremia, which was ultimately positive for rare bacteria: *M.osleonsis*. 

From 1953 to 1980, the Centers for Disease Control and Prevention has received 199 isolates that were later identified as *M.osleonsis*. The isolates consisted of specimens mostly from blood, cerebrospinal fluid, ear, nose, throat, urine, and genital secretions [13]. As our patient was found to have poor dentition upon physical exam, this may be the site of entry for M.osleonsis bacteremia. While he did have lethargy, nausea, and vomiting, he did not present with other signs and symptoms that may present in bacteremia such as fever, chills, tachypnea, tachycardia, or increased white blood cell (WBC) count. This may be due to effective cephalosporin treatment in its early stages. 

Several recent clinical trials have suggested that SARS-CoV-2 infection causes a functional decline of CD8+ T cells and natural killer cells (NK) due to continuous stimulation from the virus [14-17]. According to a study in Wuhan, China, 19 patients in the intensive care unit who were confirmed to have COVID-19 pneumonia had considerably decreased CD4+ and CD8+ T cell levels [14,17]. Another study also found decreased absolute numbers of T lymphocytes (CD4+ T cells, and CD8+ T cells) in both mild and severe cases of COVID-19 pneumonia, but to a higher degree in the severe cases. CD8+ T cells and B cells were noted to be decreased with an increase in the CD4+/CD8+ ratio in treated COVID-19 patients and were associated with poor treatment outcome [18]. CD4+ and CD8+ T cells play a crucial role in immune response against viral infections and may play a role in vaccine design and long-term immunity. CD8+ T cells release interferons (INF- γ), perforin, and granzymes to eliminate viruses, while CD4+ helper T cells enhance CD8+ cells and B cells to help them clear the viral pathogen [14-15,17].

In a recent case report, this is illustrated in a COVID-19 patient with diabetes mellitus, methicillin-sensitive *Staphylococcus aureus* (MSSA) bacteremia and osteomyelitis [17]. CD4+ and CD8+ T cell functional exhaustion may explain the reason why the patient in that case report presented with recurrent bacteremia and multi-organ infection. Despite aggressive antibiotic therapy, the patient remained with bacteremia and developed endocarditis with subsequent aortic root abscess [17]. Leukopenia was present in our patient which may be indicative of depleted T cells. Unfortunately, the CD4+/CD8+ T cell levels were not obtained for our patient, but it may suggest a way that SARS-CoV-2 infection could result in an immunosuppressed state and result in rare bacteremia, especially given that the patient was not taking any immunosuppressant medications or having immunocompromising conditions such as cancer or diabetes.

Current therapeutic treatments for COVID-19 have included immune regulation and developing antibodies for vaccines. Regulatory T cells (Tregs) offer another alternative therapy. Tregs are believed to be from the same lineage as CD4+ cells and may possibly be reduced in patients with COVID-19. Tregs play a role in regulating or suppressing other cells in the immune system. According to current literature, the level of peripheral Tregs is significantly reduced in severely affected COVID-19 patients compared to mild disease. It has been hypothesized that Tregs migrate to the lungs during tissue injury and cause peripheral reduction. Reduction in the levels of Tregs in the periphery could be associated with an overactive immune system, and in turn damaged lungs, in severely ill COVID-19 patients. One team of scientists has proposed that CD4+CD25+FoxP3+ regulatory T cell-based therapies may be beneficial. Ex vivo transplantation of polyclonal Tregs as well as allogeneic HLA-matched umbilical cord-derived Tregs have shown some positive results in COVID-19 patients [19]. Particularly, two critically ill men with coronavirus who were treated with Tregs from umbilical cord blood showed clinical improvements soon after their first infusion with Tregs, to the point that they were able to be eventually extubated with subsequent tracheostomies in place [20]. 

## Conclusions

Based on the currently published data, this is the first reported case of *M.osleonsis* bacteremia in a COVID-19 positive patient. *M.osleonsis *has been shown to affect immunocompromised patients including those with lung cancer or kidney transplants, but rarely presents with bacteremia in immunocompetent patients. This raises the question of whether the presence of a recent SARS-CoV-2 infection (or the presence of current COVID-19 antigen positivity) creates an immunocompromised state that predisposes patients to bacteremia. More studies will need to be done in order to understand those specific mechanisms of COVID-19 that allow for bacterial translocation into circulation and developing bacteremia. Current therapies for COVID-19 have varied, and further understanding of T cell regulation in COVID-19 patients may provide more robust treatments in the future. 
